# Emotional Empathic Responses to Dynamic Negative Affective Stimuli Is Gender-Dependent

**DOI:** 10.3389/fpsyg.2017.01491

**Published:** 2017-08-31

**Authors:** Kim P. C. Kuypers

**Affiliations:** Department of Neuropsychology and Psychopharmacology, Faculty of Psychology and Neuroscience, Maastricht University Maastricht, Netherlands

**Keywords:** emotional empathy, gender, dynamic stimuli, arousal, concern

## Abstract

Empathy entails the ability to recognize emotional states in others and feel for them. Since empathy does not take place in a static setting, paradigms utilizing more naturalistic, dynamic stimuli instead of static stimuli are perhaps more suited to grasp the origin of this highly complex social skill. The study was set up to test the effect of stimulus dynamics and gender on empathic responses. Participants were 80 healthy volunteers (*N* = 40 males) aged 22.5 years on average. Behavioral empathy was tested with the multifaceted empathy test, including static emotional stimuli, and the multidimensional movie empathy test (MMET), including dynamic stimuli. Findings showed emotional empathy (EE) responses were higher to negative emotional stimuli in both tasks, i.e., using static as well as dynamic stimuli. Interestingly a gender-dependent response was only seen in the MMET using dynamic stimuli. It was shown that females felt more aroused and were more concerned with people in negative affective states. It was concluded that the MMET is suited to study gender differences in EE.

## Introduction

Empathy is a multifaceted construct, encompassing a number of primary components like emotional empathy (EE), cognitive empathy, and emotion regulation. Emotional and cognitive empathy are, respectively, feeling and knowing what somebody else is feeling and thinking (Decety and Jackson, 2006; Decety, 2011; Gyurak et al., 2011). Empathy is commonly assessed by means of questionnaires like the Interpersonal Reactivity Index (IRI) (Davis, 1983) or the Empathizing/Systemizing Quotient (EQ/SQ) (Baron-Cohen et al., 2003; Claridge and McDonald, 2009), or paradigms using non-verbal static stimuli, like the Reading the Mind in the Eyes Test (Baron-Cohen et al., 2001) or the Facial Emotion Recognition Test (Kemmis et al., 2007). Previously, it has been demonstrated that females display higher self-reported empathy on questionnaires (Mestre et al., 2009; Wright and Skagerberg, 2012), and it has been shown that women are better at recognizing emotions (Kret and De Gelder, 2012). Studies into the neurobiology underlying empathy even have demonstrated gender differences in the networks involved in cognitive and EE (Christov-Moore et al., 2014). While questionnaires cover different aspects of empathy like perspective taking and empathic concern, representing cognitive and EE, respectively, it is mostly a *trait* measure. Studies aiming to assess the biological or cognitive underpinnings of *state* empathy will commonly make use of the aforementioned paradigms (e.g., Kuypers et al., 2015). The downside of these paradigms, however, is that they fail to cover the multidimensionality of empathy since the focus is on cognitive empathy (Dziobek et al., 2008). Dziobek et al. (2008) therefore designed a photo-based empathy paradigm, i.e., the Multifaceted Empathy Test (MET), assessing both cognitive and EE.

Research has also shown that the ability to perform well on empathy paradigms does not consequently mean that people are able to do this in real life (Tager-Flusberg, 2007) possibly due to the high processing demands of a complex world containing a wealth of stimuli. While capturing multiple dimensions of empathy with the MET using static stimuli of faces and/or bodies, empathy does not take place in a static ‘social vacuum’ and inclusion of more naturalistic and dynamic stimuli would aid in capturing the nature of this complex process (Dziobek, 2012; Risko et al., 2012). Emotional film fragments are an alternative way to approximate real-life circumstances. Previously it was shown that emotional film clips elicited emotional responses at the subjective and physiological level, with heart rate for example being significantly decelerated under high arousal conditions (Carvalho et al., 2012) or accelerated watching unpleasant stimuli (Fiorito and Simons, 1994). In addition film clips shown in motion increased arousal and heart-rate deceleration more compared to the same clips presented still (Detenber et al., 1998) and dynamic facial expressions were rated as more intense than static expressions (Rymarczyk et al., 2016).

The aim of the study was to investigate whether empathic responses to static emotional stimuli as used in the MET and dynamic stimuli, used in a newly designed ‘Multidimensional Movie Empathy Test (MMET)’ significantly differed. Secondly, it was aimed to study potential gender differences in behavioral (MET, MMET) and self-rated (IRI and EQ/SQ questionnaire) empathy and in physiological responses (heart rate) related to emotional stimuli (MMET). Based on previous research it was hypothesized that dynamic stimuli would elicit more EE compared to static stimuli and that females would display higher empathy levels on all measures compared to males.

## Materials and Methods

### Participants and Design

The study was conducted according to a between subject design with gender (male/female) as between subject factor. In total, 80 healthy volunteers [mean age (SD) 22.5 (3.1); 40 males] were included in the study. The male participants had a mean (SD) age of 23.2 (3.1), the female participants of 21.7 (3.0). Recruitment took place by means of flyers at Maastricht University and through ‘Sona Systems,’ a digital platform^1^ for participant recruitment. Exclusion criteria were suffering from mental illness or heart disease.

Based on comparable previous research (Codispoti et al., 2008) and a power calculation (parameters: power = 0.80; effect size *f* = medium; α = 0.05) a sample size of 80 is sufficient to detect differences between outcome measures.

### Procedure

When interested in the study, individuals were sent the information brochure of the study explaining the aims and procedures. In case of no objection, participants were included. On the test day, upon arrival at the test facilities, participants had the opportunity to ask questions about the study and its procedures. When everything was clear, they signed the informed consent.

The test session started with filling out questionnaires assessing empathy (IRI and EQ/SQ) and mood (POMS). After this, electrodes to register heart rate were attached to the chest (3-lead ECG, with a lead-II configuration) and they were connected to a laptop which registered the physiological data. In half of the group, heart rate was registered during the MMET, in the other half, heart rate was registered during another empathy task not reported here (Kuypers, unpublished). They were then seated in front of a second laptop on which they conducted the Multifaceted Empathy task first, followed by the MMET. To compensate for their time investment, participants received either a gift voucher or a participation points in case of undergraduate students who still needed to collect these points as part of the Bachelor in Psychology.

The study was approved by the Ethics Committee of Psychology of the Faculty of Psychology and Neuroscience of Maastricht University.

### Empathy Tasks

#### Multifaceted Empathy Test

The MET (Hurlemann et al., 2010) consists of 40 pictures of people conveying a complex emotional state which was positive in 50% of the pictures and negative in the other half. To assess cognitive empathy, participants had to select, out of four words, the emotion word which matched the picture. To assess EE, participants had to rate on a scale from 1 to 9 how concerned they were for the person in the picture (‘Explicit EE’) and how emotionally aroused (‘Implicit EE’) the picture made them by using the ‘Self-Assessment Manikin.’ This SAM contains a metaphor for arousal in increasing order, with five choice alternatives (Bradley and Lang, 1994). Dependent variables were the number of correct classified pictures and corresponding reaction times and the Implicit and Explicit EE ratings per valence (Dziobek et al., 2008).

#### Multidimensional Movie Empathy Test

In the MMET, 29 short movie-clips were shown with sound. The clips showing positive (*N* = 10) and negative (*N* = 9) social interaction scenes, and parts of nature documentaries (*N* = 10) were taken from the Emotional Movie Database (EMDB) developed by Carvalho et al. (2012). Carvalho et al. (2012) designed a database of non- auditory, emotion-inducing film clips including six types of fragments, i.e., social negative and horror film clips, with a clear negative content, social positive and erotic film clips, with a clear positive content, and scenery film clips (landscapes without people and animal) and object manipulation film clips with a neutral content. Since we were interested in social interactions, we chose to use the social interaction clips and take the scenery film clips as control condition. In the original database sound was left out since they wanted to produce a data base with clips to which other sounds could be added, e.g., to measure a startle response. For the purpose of the present study, sound was added since the aim was to present ecologically valid stimuli, including the auditory information of the social interaction.

The duration of the clips ranged between 35 and 50 s. They were presented in a random order with a 4 S inter-trial interval. After each clip the participant had to answer three questions. The first one was a control question, asking whether the emotional story they heard was positive, negative or neutral. Thereafter, they had to rate how concerned s/he felt for the people in the clip (scale 1–9) and how emotionally aroused the clip made them by using the Self-Assessment Manikin. Participants were instructed to choose either ‘1’ or ‘5’ in clips were no people were present (nature documentaries). The question about concern and arousal refer, respectively, to implicit and explicit EE. The dependent measures in this task are mean score of arousal and concern for positive and negative emotions and heart rate related to the clips.

Heart rate was recorded during the MMET by means of Brain Vision recorder 1.20 using a 3-lead ECG with a lead-II configuration. Collected data was preprocessed offline by means of Vision Analyzer 2.0. Preprocessing consisted of data filtering (low cut-off 20; high cut-off 70, notch: 50 Hz) after which R-peaks were automatically detected (threshold: 400 μV). After this process, a visual inspection of the data was conducted to check whether all artifacts were removed. In case of artifacts, these were manually removed. Peaks were exported to Excel where data was averaged per stimulus category to a beats per minute value (bpm). The heart rate acquired in the 4-s inter-trial intervals was averaged and served as baseline reference conform the procedure of Carvalho et al. (2012). In order to be able to compare the heart rate response related to the film clips of the three categories, a baseline % change was calculated for heart rate {e.g., for positive stories: [heart rate positive story - heart rate baseline/heart rate baseline]^∗^100}.

### Questionnaires

#### Interpersonal Reactivity Index

The IRI is a 28-item questionnaire consisting of 4 discrete seven-item scales i.e. ‘Fantasy,’ F (tendency to imaginatively transpose oneself into fictional situations), ‘Perspective-Taking,’ PT (tendency to spontaneously adopt the psychological viewpoint of others), ‘Empathic Concern,’ EC (taps the respondents’ feelings of warmth, compassion and concern for others), and ‘Personal Distress,’ PD (assesses self-oriented feelings of anxiety and discomfort resulting from tense interpersonal settings). Items are rated on a 5-points scale (1–5) with a maximum score of 35 per scale. The first two scales are a measure of Cognitive Empathy; the two latter a measure of EE (Davis, 1983).

#### The Empathizing (EQ)-Systemizing Quotient (SQ)

The EQ-SQ questionnaire consists of 120 statements in a forced choice format (i.e., strongly agree; slightly agree; slightly disagree; strongly disagree). Half of the items form the SQ, the other half the EQ. Both scales contain 20 filler items and 40 ‘real’ items tapering the construct of interest, i.e., respectively, ‘Systemizing’ (= the drive to analyze systems or construct systems) and ‘Empathizing’ (= the drive to identify mental states and respond to those with an appropriate emotion). The maximum score on both scales is 80. Previously it has been shown that normal male adults score higher on the SQ and lower on the EQ compared to women (Baron-Cohen et al., 2003; Claridge and McDonald, 2009).

#### The Profile of Mood States

The Profile of Mood States (POMS) (de Wit et al., 2002) is a self-assessment mood questionnaire with 72 five point-Likert scale items, representing eight mood states; i.e., Anxiety, Depression, Anger, Vigor, Fatigue, Confusion, Friendliness and Elation. Two extra scales are derived, i.e., Arousal [(Anxiety + Vigor) – (Fatigue + Confusion)] and Positive mood (Elation – Depression). The participant had to indicate to what extent these items were representing his/her mood.

### Statistical Analyses

Data of the MET and MMET entered a General Linear Model (GLM) mixed ANOVA with Gender as between subject factor (two levels) and Valence as within subject factor (three levels: positive, negative, neutral for MMET Arousal and MMET heart rate; and two levels for MET and MMET Concern) (SPSS version 24.0). In case of main effects, Bonferroni-corrected pairwise comparisons were conducted; in case of interaction effects, *t*-tests were run to test the origin of the interaction. Since previous research has shown that heart rate was decelerated more under high arousal conditions (Carvalho et al., 2012), arousal ratings on the MMET were correlated (Pearson) with heart rate.

In order to test whether EE differed between MET and MMET, arousal and concern ratings entered two separate GLM RM ANOVAs with Test (two levels, MET and MMET), and Valence (two levels, positive and negative) as within subject factors.

Mood (POMS) and empathy (IRI, EQ/SQ) questionnaire data entered three separate multivariate ANOVAs with Gender as fixed factor.

The alpha criterion level of statistical significance for all analyses was set at *p* = 0.05; partial eta^2^ (η^2^) is reported in case of significant effects to demonstrate the effect’s magnitude (0.01: small, 0.06: moderate; 0.14: large).

## Results

### Behavioral Measures

#### Multifaceted Empathy Test (MET)

**FIGURE 1:**
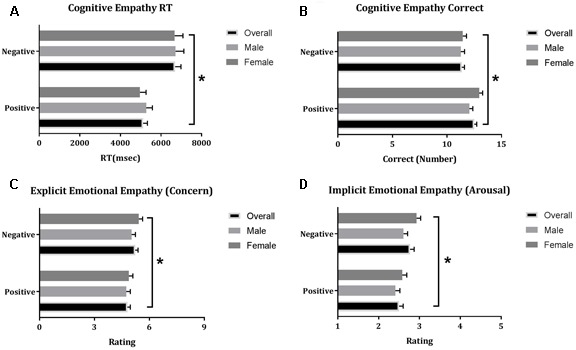
Mean (±SE) of reaction times related to correct recognized emotions **(A)** and the number of correct recognized emotions **(B)** and ratings of Concern **(C)** and Arousal **(D)** in the Multifaceted Empathy Test; ^∗^indicates statistical significance at *p* = 0.05.

Repeated measures GLM revealed a main effect of Valence on dependent variables related to Cognitive Empathy, i.e., Number of correct recognized (*F*_1,78_ = 12.36, *p* = 0.001, η^2^ = 0.14) and corresponding Reaction Time (*F*_1,78_ = 52.26, *p <* 0.001, η^2^ = 0.41) and dependent variables related to EE, i.e., Concern (*F*_1,78_ = 26.30, *p* < 0.001, η^2^ = 0.25) and Arousal (*F*_1,78_ = 40.14, *p* < 0.001, η^2^ = 0.34). Participants were faster at recognizing positive emotions and they recognized more positive emotions correctly compared to negative emotions whereas they were more aroused by negative emotions and more concerned with people depicting negative emotions (**Figure 1**).

There was no main effect of Gender or interaction effect between Gender and Valence on parameters of the MET.

#### Multidimensional Movie Empathy Test (MMET)

On average (±SD) 97% (5.6) of the positive stories, 98% (4.6) of the negative stories, and 98% (7.4) of the neutral stories were classified in the correct emotion category.

**FIGURE 2:**
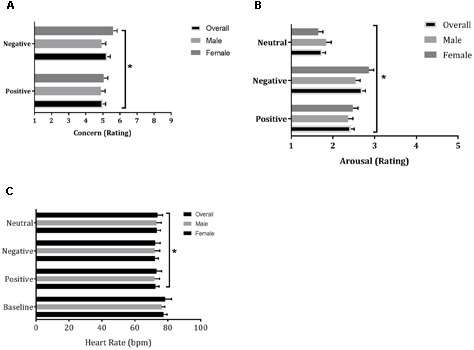
Mean (±SE) ratings of Concern **(A)** and Arousal **(B)** in response to positive and negative clips and heart rate (bpm) **(C)** during baseline and positive, negative, and neutral movies in the Multidimensional Movie Empathy Test; ^∗^indicates statistical significant main effect at *p* = 0.05.

Repeated measures GLM revealed a main effect of Valence (*F*_1,78_ = 10.15, *p* = 0.002, η^2^ = 0.11) on Concern, i.e., more concern was reported when watching negative emotion movie clips compared to positive (**Figure 2A**). Analysis also revealed a Valence by Gender interaction effect (*F*_1,78_ = 7.40, *p* = 0.008, η^2^ = 0.09) on Concern. Additional analyses showed that this was caused by females feeling significantly (*t*_39_ = -4.29; *p* < 0.001) more concern when presented with negative movie clips compared to positive movie clips. This difference was absent in males (*t*_39_ = -0.32; *p* = 0.75); when comparing concern between males and females, the gender difference approached statistical significance for the negative clips (*t*_78_ = -1.89; *p* < 0.06), while this difference was absent for the positive clips (*t*_78_ = -0.45; *p* = 0.65).

General linear model RM ANOVA revealed a main effect of Valence (*F*_1,156_ = 72.62, *p* < 0.001, η^2^ = 0.48) on Arousal. Pairwise comparisons showed that arousal levels in response to positive, negative and neutral clips all differed significantly (*p* < 0.001), with experienced arousal being highest for negative clips, lowest for neutral clips and intermediate for positive clips (**Figure 2B**). In addition, an interaction between Valence and Gender (*F*_1,78_ = 4.20, *p* = 0.04, η^2^ = 0.05) was found on Arousal. Additional analyses revealed that the interaction effect was due to a higher arousal rating by females compared to males on negative movie clips (*t*_78_ = -1.99; *p* = 0.05).

Analysis revealed a main effect of Valence (*F*_2,78_ = 4.03, *p* = 0.02, η^2^ = 0.10) on heart rate. The decrease in heart rate relative to baseline was larger in the negative movie clip condition compared to the decrease in heart rate in the neutral movie clip condition (*p* = 0.006). There was no main effect of Gender or an interaction effect of Valence by Gender on heart rate (**Figure 2C**).

Heart rate and Arousal ratings on the MMET did not correlate for any of the emotion category clips.

#### Emotional Empathy in MET and MMET

General linear model RM Analyses showed no main effect of Test on EE ratings on the MET and MMET, i.e., concern (*F*_1,79_ = 1.35, *p* = 0.25, η^2^ = 0.02) and arousal (*F*_1,79_ = 2.10, *p* = 0.15, η^2^ = 0.03) ratings were similar on both tests. A main effect of Valence demonstrated concern (*F*_1,79_ = 22.33, *p* < 0.001, η^2^ = 0.22) and arousal (*F*_1,79_ = 42.32, *p* < 0.001, η^2^ = 0.35) ratings were higher when presented with negative emotion stimuli compared to positive stimuli. There was no Test by Valence interaction for Concern (*F*_1,79_ = 1.60, *p* = 0.21, η^2^ = 0.02) or Arousal (*F*_1,79_ = 0.01, *p* = 0.92, η^2^ = 0.00).

### Questionnaires

#### Interpersonal Reactivity Index

**FIGURE 3:**
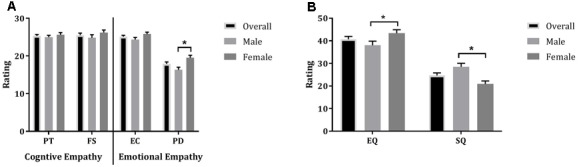
Mean (±SE) of ratings of males, females, and both groups together (overall) on subscales of the Interpersonal Reactivity Index **(A)** and the Empathizing-Systemizing (EQ/SQ) questionnaire **(B)**; PT, Perspective Taking; FS, Fantasy Scale; EC, Empathic Concern; PD, Personal Distress; ^∗^indicates statistical significance at *p* = 0.05.

Multivariate ANOVA revealed no statistically significant differences between males and females on the two cognitive empathy scales, i.e., Perspective Taking and Fantasy Scale. Males and females statistically differed on one of the two EE scales, i.e., Personal Distress (*F*_1,78_ = 10.45, *p* = 0.002, η^2^ = 0.12); it was shown that females scored higher on ‘personal distress’ compared to males (**Figure 3A**).

#### The Empathizing (EQ)-Systemizing Quotient (SQ)

Analyses revealed that males scored higher on the systemizing quotient compared to women (*F*_1,78_ = 13.84, *p* < 0.001, η^2^ = 0.15) and that women scored higher on the empathizing quotient compared to men (*F*_1,78_ = 5.06, *p* = 0.03, η^2^ = 0.06) (**Figure 3B**).

#### The Profile of Mood States

**FIGURE 4:**
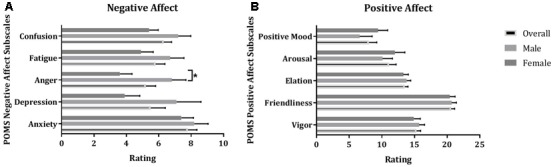
Mean (±SE) of ratings of males, females, and both groups together (overall) on Negative **(A)** and Positive **(B)** Affect scales of the Profile of Moods States; ^∗^indicates statistical significance at *p* = 0.05.

Analysis revealed no statistical differences between males and females on the Positive Affect scales of the POMS; a statistically significant difference between males and females was shown on one Negative Affect scale, i.e., Anger (*F*_1,78_ = 7.68, *p* = 0.007, η^2^ = 0.09). Males reported higher levels of anger compared to women on a test day, before conducting the empathy tests (**Figure 4**).

## Discussion

The aim of the present study was to investigate whether empathic responses to static and dynamic emotional stimuli differ or show similarities and whether there are gender differences in empathic responses on subjective, behavioral and physiological measures. It was hypothesized that dynamic stimuli would elicit more EE compared to static stimuli and that females would display higher empathy levels on all measures compared to males. Findings showed that the largeness of the empathic response was influenced by valence but not by ‘dynamics’ of the stimulus, i.e., arousal and concern were rated higher when confronted with negative emotional stimuli compared to positive emotional stimuli in both the MET and MMET, using static and dynamic stimuli, respectively. Interestingly, a gender-dependent response was only seen in the MMET, using dynamic stimuli, i.e., females expressed to feel higher levels of arousal and they were more concerned with people when confronted with negative emotional scenes. This gender difference was not demonstrated at the physiological level, i.e., when watching negative emotion movie clips the heart rate of both males and females decelerated more when watching negative emotional scenes, compared to positive movie clips and baseline heart rate. Physiological and behavioral measures of arousal were not associated in the MMET. Empathy trait questionnaires showed that females scored higher on ‘Personal Distress’ and displayed a higher ‘empathizing quotient’ compared to males, whereas the latter group had a higher ‘systemizing quotient’ compared to females.

The absence of differences in empathic responding between the tasks showing static and dynamic stimuli was opposite to what was hypothesized. Previously it was shown that moving stimuli elicited more arousal compared to static emotional stimuli (Detenber et al., 1998) and negative (angry) faces were judged as more intense than positive (happy) faces when confronted with dynamic compared to static stimuli (Biele and Grabowska, 2006). This difference could lie in the stimuli that were used, i.e., whereas the present study used pictures of faces in the MET and film fragments in the MMET, the other studies used static and dynamic versions of the same stimulus, e.g., either still or moving pictures of faces or film fragments. To exclude this possibility it would be good to include variations in dynamics in both tests, i.e., the MET and MMET in future studies.

Interestingly a task-selective gender difference in EE was demonstrated in the behavioral response to dynamic stimuli, i.e., females demonstrated higher EE compared to males but only when confronted with negative dynamic stimuli. Previously it was shown that females recruit brain areas containing mirror–neurons more than males when focusing on the emotion they experience when confronted with other’s negative emotions (fear, anger) and when thinking about other’s emotions. It was suggested that this difference in brain activity might underlie a higher level of emotional contagion in females (Schulte-Rüther et al., 2008) which would explain the higher level of arousal and concern, elicited in females in response to negative emotions. It is apparent from the current findings that the MMET is suited to study gender differences in EE. However, a limitation is that it only assesses one dimension, i.e., EE, in contrast to the MET. A complimentary paradigm in future studies, also using dynamic stimuli could be the Movie for the Assessment of Social Cognition, which assesses cognitive empathy and perspective taking (Dziobek et al., 2006).

Heart rate revealed a valence-specific response pattern on the MMET, i.e., heart rate decelerated when viewing negative emotional movie clips compared to neutral clips in both males and females. These data are in line with a previous study (Anttonen and Surakka, 2005) using stimuli with similar characteristics as the stimuli used in the present study, i.e., segments with auditory, visual and audiovisual stimuli, though they were shorter (6 s). Negative affect stimuli were linked to a larger deceleration in heart rate (Anttonen and Surakka, 2005). Another study explained the deceleration of heart rate by an increase in allocation of attention when presented with high arousing stimuli (Detenber et al., 1998). It is known that although physiological changes may reflect discrete emotional responses, they can also be influenced by a wide range of non-emotional factors (e.g., attention, cognition, physical activity, or extraneous external stimuli) (Eisenberg and Fabes, 1990). Other physiological measures like skin conductance might be more sensitive to the (gender-specific) behavioral changes on emotional paradigms since this measure has been shown to increase with reported affective arousal. (Greenwald et al., 1989; Lang et al., 1993). In addition, electromyography (EMG) recordings have previously also been shown to differentiate between emotional states, e.g., with different facial EMG response patterns being evoked by happy and angry facial expressions (Dimberg, 1982) and women exhibiting larger EMG responses to dynamic happiness stimuli compared to static stimuli and the response of males (Rymarczyk et al., 2016).

In line with previous findings, females scored higher on the empathizing quotient compared to males and lower on systemizing (Wakabayashi et al., 2007; Wright and Skagerberg, 2012). However, in contrast to most findings of previous studies, where gender differences were revealed for all IRI scales (Davis, 1983; De Corte et al., 2007), females only differed from males on one subscale, i.e., Personal Distress, with females scoring higher. The same pattern though has been shown in the past, in a sample of male and female nurses (Becker and Sands, 1988).

In sum, the present study shows that the MMET using dynamic emotional stimuli elicits the same behavioral response as the MET, using static stimuli. In addition, it was shown that dynamic stimuli provoke gender-dependent reactions, which makes this task suited to study gender differences in EE. Future studies should, however, include additional empathy measures with dynamic stimuli assessing cognitive empathy, in order to cover the spectrum of empathic processes.

## Ethics Statement

This study was carried out in accordance with the recommendations of ‘the Ethics Committee of Psychology of the Faculty of Psychology and Neuroscience of Maastricht University’ with written informed consent from all subjects. All subjects gave written informed consent in accordance with the Declaration of Helsinki. The protocol was approved by the ‘the Ethics Committee of Psychology of the Faculty of Psychology and Neuroscience of Maastricht University.’

## Author Contributions

KK has designed the study protocol, supervised the data collection, analyzed the data and written the manuscript.

## Conflict of Interest Statement

The author declares that the research was conducted in the absence of any commercial or financial relationships that could be construed as a potential conflict of interest.

## Footnotes

1 maastricht-fpn.sona-systems.com
